# Hierarchical nickel carbonate hydroxide nanostructures for photocatalytic hydrogen evolution from water splitting†

**DOI:** 10.1039/d3ma00977g

**Published:** 2024-02-27

**Authors:** Parisa Talebi, Rossella Greco, Takashi Yamamoto, Mahdiyeh Zeynali, Saeid Asgharizadeh, Wei Cao

**Affiliations:** a Nano and Molecular Systems Research Unit, University of Oulu FIN-90014 Finland Rossella.Greco@oulu.fi; b Department of Science and Technology, Tokushima University Tokushima 770-8506 Japan; c Faculty of Physics, University of Tabriz Tabriz 5166616-471 Iran

## Abstract

Metal carbonate hydroxides have emerged as novel and promising candidates for water splitting due to their good electrochemical properties and eco-friendly features. In this study, the hierarchical mesoporous structure of nickel carbonate hydroxide hydrate (Ni_2_(CO_3_)(OH)_2_·4H_2_O) was synthesized by a one-pot facile hydrothermal method. It demonstrated photocatalytic properties for the first time, exhibiting a hydrogen evolution reaction yield of 10 μmol g^−1^ h^−1^ under white light irradiation with a nominal power of 0.495 W. This facile synthesis strategy and the good photocatalytic properties indicate that nickel carbonate hydroxide is a promising material for application in photocatalytic hydrogen evolution.

## Abstract 2

Metal carbonate hydroxides have emerged as novel and promising candidates for water splitting due to their good electrochemical properties and eco-friendly features.

## Graphical abstract

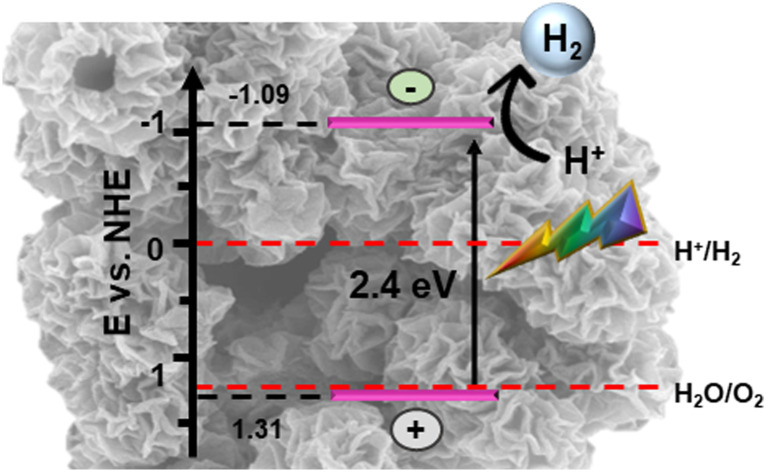

## Introduction

It is well-known that photocatalytic performances are strongly dependent on the structural and morphological features of the materials used as photocatalysts. Among them, hierarchical materials have shown great potential for various photocatalytic applications such as hydrogen (H_2_) evolution, dye degradation, and CO_2_ reduction.^1^ This is primarily related to several advantageous features of these materials, including enhanced molecular diffusion/transfer, improved light harvesting, high surface-to-volume ratio, abundant transport paths for small organic molecules, easy separation, and good recyclability.^2,3^ Hierarchical micro-/nanostructures are assemblies of 1D or 2D nanoscale building blocks such as nanoparticles, nanorods, nanoribbons, nanosheets, *etc*.^4^ However, the synthesis of hierarchical materials typically involves the use of expensive and sometimes toxic templates or surfactants to facilitate the assembly process.^1,5^ Therefore, a method for preparing hierarchical structures without any polymer or similar additives is highly desired, thereby opening up new possibilities for efficient and sustainable photocatalytic applications.

Many articles have reported transition metals-based (Cu, Fe, Co, Ni, and Mn) materials with hierarchical nanostructures including oxides, nitrides, sulfides, carbonates, and hydroxides for water splitting owing to their suitable band gap and excellent stability.^6,7^ Among all the attractive materials, layered metal carbonate hydroxides (MCHs) have shown great potential for water splitting due to their proper redox potentials for water and high accessibility to electrolytes.^8,9^ MCHs are expressed by the general formula M_*x*_(OH)_*y*_CO_3_·*n*H_2_O, where M is the metal ion in the +2 oxidation state.^10^ These carbonate hydroxide materials demonstrate good electrochemical properties due to the CO_3_^2−^ hydrophilic nature, which enhances the wettability of the electrode surface.^11,12^ There are some reports for photocatalytic O_2_ evolution using Co-based MCHs;^13^ however, MCHs have not been explored for the photocatalytic hydrogen evolution reaction (HER) yet. Ni-based MCHs possess the optimal potential for water reduction to H_2_, but their synthesis still represents a limiting factor for applying them in energy applications. For all these reasons, Ni-based MCHs are a potential research gap and an opportunity to explore the photocatalytic performance of MCHs for the HER.

In our previous works, we reported the role of nickel carbonate in commercial Ni-based plasmonic core@shell hybrid nanostructures for photocatalytic HER from water splitting.^14,15^ In this work, hierarchical nickel carbonate hydroxide was synthesized *via* the hydrothermal method and used as a photocatalyst for the HER from water splitting in the absence of any sacrificial agent, which usually leads to side products with unknown toxicity.^16^ The crystal structures, chemical compositions, and morphologies of this material were investigated in detail through X-ray diffraction (XRD), diffuse reflectance infrared Fourier transform spectroscopy (DRIFT), X-ray photoelectron (XPS) spectroscopies, and scanning electron microscopy (SEM). The material exhibited favorable photocatalytic activity, indicating the potential of MCHs as promising candidates for high-performance photocatalytic water splitting. These findings provide valuable insights for further exploration and optimization of MCHs in photocatalysis.

## Experimental

**Scheme 1:**
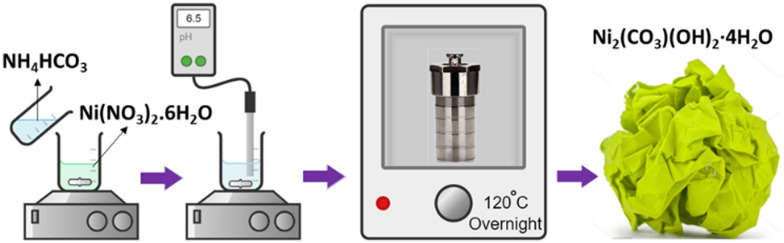
Hydrothermal synthetic procedure followed for the preparation of NCH.

Nickel carbonate hydroxide was synthesized by a hydrothermal method (Scheme 1).^17^ Typically, 2.5 mmol nickel nitrate hydrate (Ni(NO_3_)_2_·6H_2_O) (99%, Sigma-Aldrich) was dissolved in 200 mL deionized (DI) water, while 5 mmol ammonium bicarbonate NH_4_HCO_3_ (99%, Sigma-Aldrich) in 50 ml DI water. The two solutions were mixed and stirred for 10 minutes. To achieve a pH ≈ 6.5, citric acid (0.1 g ml^−1^ in DI water) was added to the solution while stirring. The resulting solution was transferred to a Teflon-lined stainless-steel autoclave and hydrothermally treated at 120 °C overnight. After the autoclave was naturally cooled down to room temperature, the sample was taken out, washed with DI water several times, and dried under vacuum overnight. The obtained product was labeled as NCH. The possible chemical reactions involved in the aforementioned preparation process are depicted below.Ni(NO_3_)_2_·6H_2_O → Ni^2+^ (aq.) + 2NO_3_^−^ + 6H_2_ONH_4_HCO_3_ → NH_3_ + CO_2_ + H_2_ONH_3_ + H_2_O → NH_4_^+^ + OH^−^CO_2_ + 2OH^−^ → 2H^+^ + CO_3_^2−^2Ni^2+^ + CO_3_^2−^ + 2OH^−^ + 4H_2_O → Ni_2_(CO_3_)(OH)_2_·4H_2_O

## Results and discussion

**Fig. 1:**
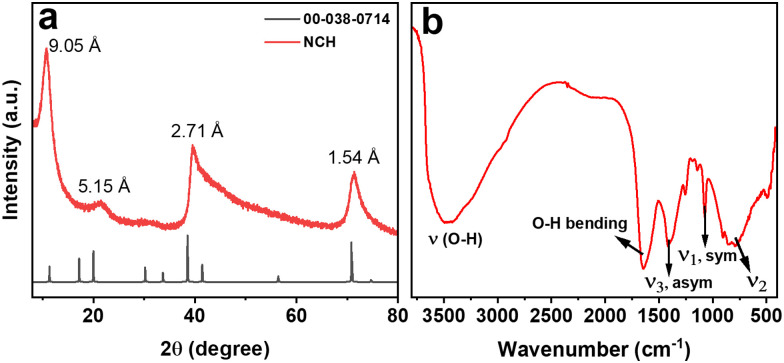
(a) XRD, and (b) DRIFT of NCH.

To identify the crystal structure and phase purity of the NCH, powder XRD was employed. The XRD pattern of the NCH obtained after the hydrothermal synthesis is shown in Fig. 1a. The diffraction pattern of the sample corresponds to a carbonate hydroxide structure, specifically Ni_2_(CO_3_)(OH)_2_·4H_2_O (JCPDS NO. 00-038-0714). The peaks in the pattern exhibit a broad full width at half maximum (FWHM), indicating a nanostructured morphology of the sample. The peaks positions and corresponding d-spacing values are observed at 11.3° (*d* = 9.05 Å), 19.9° (*d* = 5.1 Å), 38.5° (*d* = 2.7 Å), and 70.8° (*d* = 1.54 Å). Compared to JCPDS NO. 00-038-0714, the peaks are shifted to higher angles, possibly due to a stretched crystal structure. The lack of impurity peaks in Fig. 1a confirms the purity of the sample. Even though the sample might not look extremely crystalline, it represents one of the few examples in the literature where a quasi-crystalline phase could be obtained for Ni_2_(CO_3_)(OH)_2_·4H_2_O.^18^ Density functional theory (DFT) calculations could help us in determining the crystal structure of the material, as shown in Fig. S1 (ESI†), and we could determine the structure to be monoclinic with *a* = 3.08044, *b* = 12.1303 and *c* = 9.59443. Additionally, we confirmed the absence of reactants comparing the XRD pattern of NCH with the pattern of NH_4_HCO_3_ and Ni(NO_3_)_2_·6H_2_O (Fig. S2, ESI†), which were used as precursors in the synthesis of NCH.

Carbonates are commonly studied by DRIFT to unveil the presence of C–O bonds; hence, DRIFT measurements were used to further explore the composition of the synthesized NCH nanostructured material. The DRIFT spectrum of NCH was obtained in the range from 400 to 4000 cm^−1^, as shown in Fig. 1b. The broad peak at 3450 cm^−1^ belongs to O–H stretching vibration, which is a feature of hydrogen band groups and molecular water. Additionally, a narrow band at 1634 cm^−1^ suggests the bending mode of water molecules. The spectrum also exhibits vibrations related to the CO_3_^2−^ group, which are distinctive for carbonates. Specifically, vibrations corresponding to the asymmetrical stretching (*ν*_3_) and symmetric stretching (*ν*_1_) of the carbonate group were observed at 1411 and 1067 cm^−1^, respectively. The out-of-plane bending absorption (*ν*_2_) of the carbonate group was detected at approximately 850 cm^−1^.^19^ Furthermore, a band below 600 cm^−1^ can be attributed to the bending vibration of the Ni–O bond.^20^ DRIFT analysis further affirms the formation of the Ni_2_(CO_3_)(OH)_2_·4H_2_O phase by verifying the presence of carbonate groups.

**Fig. 2:**
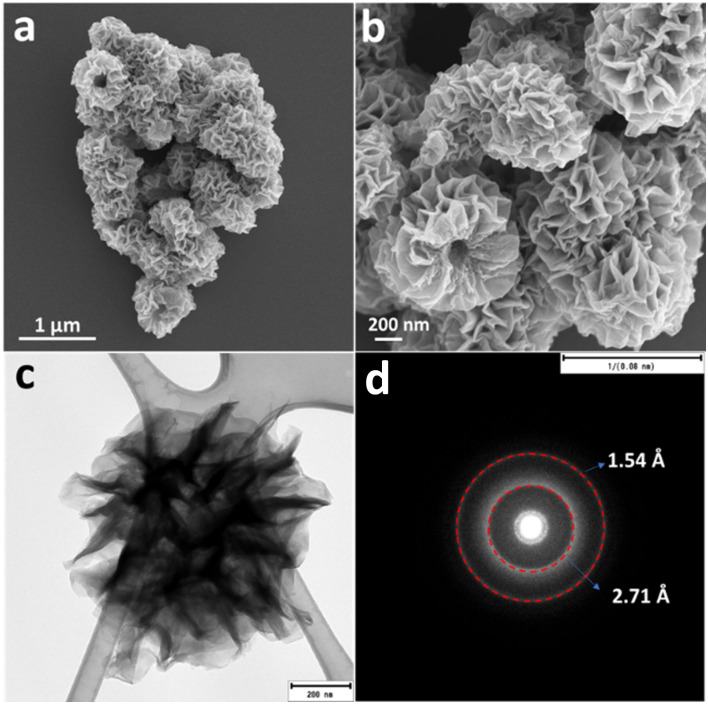
(a) and (b) FESEM, (c) TEM image, and (d) SEAD pattern of NCH.

The morphology of the sample was characterized by FESEM and Fig. 2(a) and (b) presents representative images for NCH at different magnifications. As evident, a hierarchical nanostructured architecture is well-formed by very thin sheets (Fig. S3, ESI†) and with an average diameter of around 500 nm. Several attempts to obtain a higher resolution image in high-resolution transmission electron microscopy (HRTEM) were made but failed to give clear images due to radiation/particle damage of the hydroxides from the powerful electron beam. Despite this, more detailed structural information of the hierarchical NCH was revealed using TEM. Fig. 2c presents the TEM image of an individual microsphere, which is a dandelion-like sphere, with a diameter in the range of 600 nm. The selected area electron diffraction (SAED) pattern (Fig. 2d) clearly reveals the polycrystallinity of the NCH with the *d*-spacing of 2.7 Å and 1.54 Å, which confirms the XRD results. The two-dimensional feature of this material is extremely relevant for photocatalytic applications, considering the possibility of obtaining a suitable bandgap for visible light-mediated processes.^20^

The elemental mapping analysis of NCH (Fig. S4a, ESI†) confirms the presence of Ni, O, and C. Each element is uniformly dispersed throughout the whole region of the hierarchical nanostructured spheres. Fig. S4b (ESI†) shows the energy dispersive spectrometer (EDS) analysis of NCH. While the exact quantification of these elements was challenging, the estimated weight proportions are approximately 59.99% for Ni, 10.83% for C, and 29.18% for O. The relatively high amounts of carbon and oxygen can be attributed to the presence of CO_3_^2−^, which is formed through the hydrolysis of ammonium bicarbonate, and it is adsorbed on the surface. Therefore, it is inferred that the NCH compound phase consists of Ni^2+^, OH^−^, and CO_3_^2−^ ions. The elemental analysis results showed 10.1 wt% of C in the NCH sample, which confirms the EDS result for C (10.83 wt%) and gives more insights into the composition of the material.

Considering the texture observed by SEM, the porosity of the material was evaluated by N_2_ adsorption–desorption isotherm measurement. The results of these measurements are presented in Fig. S5 (ESI†). The Brunauer–Emmett–Teller (BET) surface area of NCH was found to be 4.16 m^2^ g^−1^. The N_2_ physisorption isotherm of NCH showed a type-IV isotherm and it is categorized as an H3 type hysteresis loop according to the IUPAC classification, which is typical of mesoporous materials.^21^ The pore size distribution curve in the inset in Fig. S5 (ESI†), obtained using the BJH method, confirmed that the majority of the pore diameters fell within the mesoporous range of 2–20 nm.

**Fig. 3:**
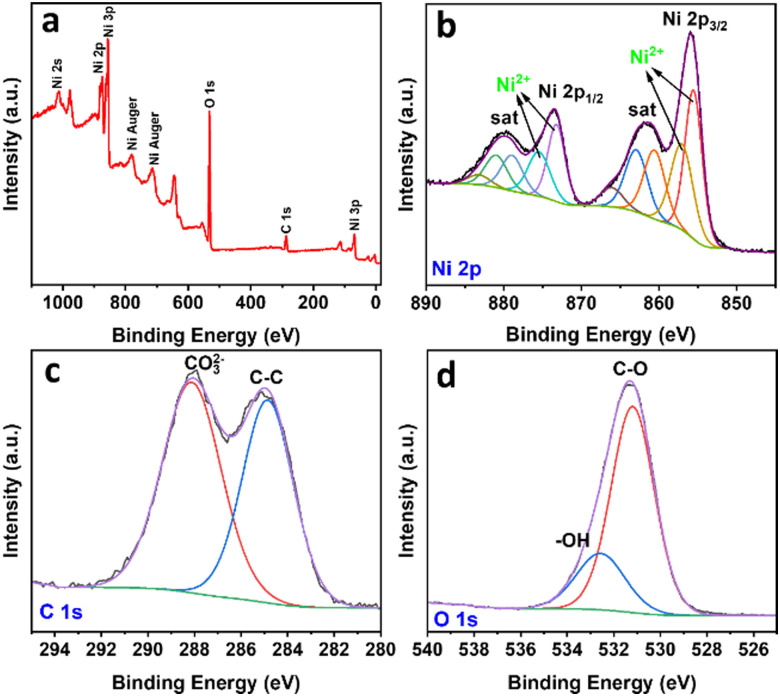
XPS spectrum of NCH: (a) survey, (b) Ni 2p, (c) C 1s, and (d) O 1s.

To further reveal the properties of the synthesized material, the sample was characterized by XPS, and the results are shown in Fig. 3(a)–(d). The XPS survey spectrum confirmed the existence of Ni, C, and O as the main elements, in good agreement with the earlier discussion. In the Ni 2p spectrum, the Ni 2p_3/2_ peaks at 855.8 eV and 857.1 eV can be attributed to Ni^2+^ species in Ni(OH)_2_ and NiCO_3_. In the high-resolution XPS, the Ni 2p spectrum demonstrates the peak splitting energy of 17.6 eV between its Ni 2p_3/2_ and Ni 2p_1/2_, which corresponds to the spin–orbit coupling. Moreover, two peaks at 860.8 eV and 879.1 eV can be signed to shake-up satellites (marked as “sat”).^22^ In the C 1s spectrum, the peak at 284.8 eV is attributable to adventitious carbon species, whereas the peak at 288.6 eV corresponds to the carbonate species. In the O 1s spectrum, the strong signal at 531.2 eV can be assigned to C–O and the peak at 532.6 eV belongs to bounded hydroxide groups (–OH).^23^ The XPS results confirmed the presence of CO_3_^2−^ and OH^−^ groups and agreed with the EDX, elemental analysis, and DRIFT results.

**Fig. 4:**
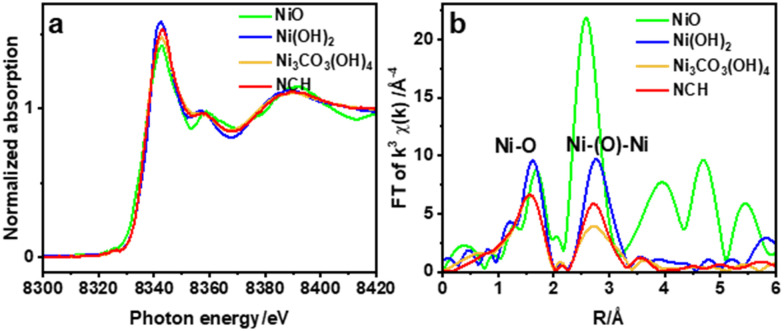
Ni K-edge (a) XANES, and (b) Fourier transforms of EXAFS of NCH and commercial samples.

X-ray absorption near edge structure (XANES) and extended X-ray absorption fine structure (EXAFS) spectroscopies were used to study the chemical composition and structure of the nanostructured NCH. Fig. 4a shows the Ni K-edge XANES spectra of NCH samples, as well as of Ni(OH)_2_, NiO, and commercial Ni_3_CO_3_(OH)_4_·4H_2_O (FUJIFILM Wako) for comparison. It is clearly visible from the spectra in Fig. 4a that all the samples exhibit three distinct features, (i) a weak pre-edge at 8325.0 eV corresponding to the dipole-forbidden 1s → 3d transition, (ii) a sharp feature at 8342.5 eV, and (iii) a post-edge at 8358.0 eV due to the multiple scattering.^24^ The main peak with a low energy shoulder of the absorption edge corresponds to the 1s → 4p electron transition and ‘shape-resonances’ of the metal atom environment.^25^ Chemical composition and unoccupied electronic states denoted in the XANES region show that the synthesized NCH is very much the same as the commercial Ni_3_CO_3_(OH)_4_·4H_2_O, consistent with the phases identified by XRD and EDS. Additionally, the first derivative spectra of Fig. 4a are shown in Fig. S7 (ESI†), which revealed that the valence states of nickel in commercial Ni_3_CO_3_(OH)_4_ and NCH are almost the same but different from those in (hydr)oxides. The non-linear least-square EXAFS fit results for the Ni coordination environments are displayed in Table S1 and Fig. S6 (ESI†). The interatomic distance of NCH is 2.04 Å and 3.10 Å (Table S1, ESI†) corresponding to Ni–O, and Ni–Ni, respectively, which are close to the values obtained by DFT (1.89 Å and 3.06 Å). The Fourier transform of EXAFS oscillations observed at the Ni K-edge of the studied sample is also shown in Fig. 4b. The broadening of the peaks in the EXAFS curves accounted for the thermal and structural disorder. In general, the EXAFS analysis data provided additional confirmation that all nickel was in the Ni^2+^ state in the NCH.^26^ The exact crystal phase of metal carbonate hydroxides is ambiguous and requires further investigation.^27,28^ Nevertheless, the coordination number ∼6 obtained by EXAFS for Ni–O (Table S1, ESI†) might imply an octahedral configuration of Ni. Note that the spectral configuration of *k*^3^-weighted EXAFS of NCH and the interatomic distance of the Ni–Ni pair are quite different from those of NiO, as shown in Fig. S4 and Table S1 (ESI†).

**Fig. 5:**
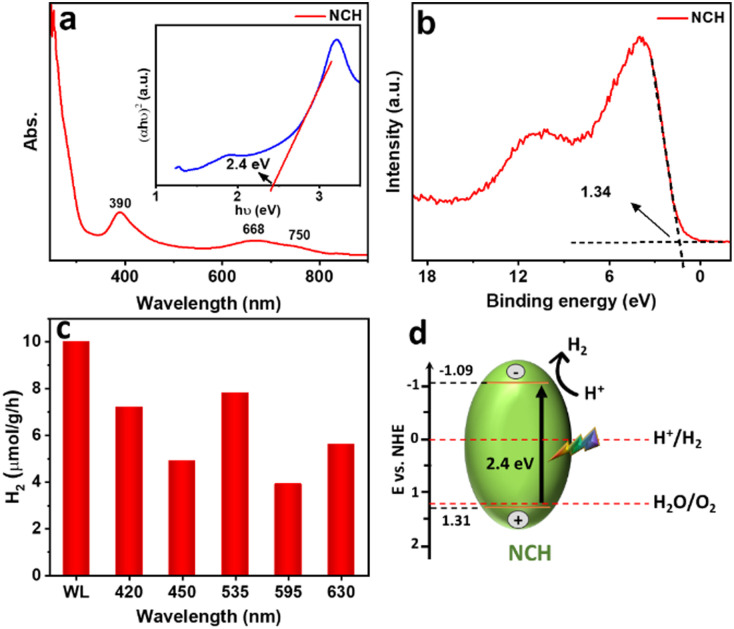
(a) UV-vis absorbance spectrum and Tauc plot (inserted image), (b) VB XPS of NCH, (c) hydrogen evolution under white light and at different wavelengths in the range of 420–630 nm using NCH as the photocatalyst, and (d) schematic representation of the proposed photocatalytic mechanism.

Fig. 5a shows the UV-vis absorption spectrum, which is used to study the optical properties of the synthesized sample dispersed in DI water in the wavelength range of 250–900 nm. As a first-row transition metal, Ni^2+^ has a d^8^ electronic configuration, as shown by XANES, and the ground state (3A_2g_) of the Ni ion in an octahedral field displays two spin-allowed transitions. The first peak at 390 nm belongs to the 3A_2g_ to 3T_1g_(P) transition range and the 3A_2g_ to 3T_1g_ transition band in the 550–800 nm range which is split into two shoulders at 668 nm and 750 nm, due to spin–orbit coupling.^29^ The Tauc plot in the inset of Fig. 5a revealed that the optical band gap energy of the material is 2.4 eV, confirming the visible light activity of the material. The value obtained for the band gap is close to the previously reported bandgap of NiCO_3_ (2.52 eV).^30^ To obtain more insight and a better understanding of the electronic structure in NCH, valence band (VB) XPS was performed (Fig. 5b). The sample showed a VB with the edge of the maximum energy at approximately 1.34 eV *versus* the Fermi level. Considering the optical bandgap, the conduction band (CB) minimum of the NCH would occur at −1.06 eV. Thus, the CB and VB of NCH are located at −1.09 and 1.31 eV with respect to the normal hydrogen electrode (NHE).^31^ Furthermore, to have a complete set of knowledge about this material, the electronic band structure and DOS were calculated. The electronic band gap obtained by the band structure and DOS is 2.25 eV (Fig. S8, ESI†).

For the photocatalytic HER, this sample was dispersed in DI water and the photocatalytic performance was recorded and analyzed. Control experiments were carried out for commercial Ni_3_CO_3_(OH)_4_·4H_2_O (Fig. S9, ESI†) and Ni(OH)_2_ (Fig. S10, ESI†) and compared to NCH under white light, as shown in Fig. S12a (ESI†). The HER using Ni_3_CO_3_(OH)_4_·4H_2_O and Ni(OH)_2_ reaches <1 μmol g^−1^ h^−1^ which is much less than NCH (∼10 μmol g^−1^ h^−1^), showing the potential of NCH in photocatalytic applications compared to other Ni-based compounds in the absence of any sacrificial agent. A possible reason for these results is also related to the less homogeneous morphology and the bigger dimensionality of Ni_3_CO_3_(OH)_4_·4H_2_O and Ni(OH)_2_, which are definitely limiting factors for an optimal catalyst, which is well-known to have better performance in the case of smaller surface areas. Another reason, as shown in Fig. S11 (ESI†), is that Ni_3_CO_3_(OH)_4_ and Ni(OH)_2_ exhibit lower light absorption and a larger bandgap in comparison to NCH. As a result, these materials do not demonstrate strong photocatalytic activity under visible light. Additionally, the potentiality of this material was confirmed by stability tests (Fig. S12b, ESI†), where we could see only a slight decrease of the H_2_ production in the 2nd and 3rd cycles. Fig. 5c shows the H_2_ production rate for the synthesized sample under white light illumination and in the range of 420 nm to 630 nm for NCH. The highest observed HER performance is ∼8 μmol g^−1^ h^−1^ at the excitation wavelength of 535 nm (2.3 eV), which is close to the bandgap of the material (2.4 eV). Fig. 5d presents a schematic band diagram and possible hydrogen generation mechanism of the sample, which is proposed based on the combined results of the UV-vis absorption and VB XPS results. The CB of NCH is at a more negative potential compared to the potential required for H^+^ reduction. This suggests that the photogenerated electrons possess enough energy to effectively reduce H^+^ and produce H_2_. During photocatalysis, electron–hole (e^−^–h^+^) pairs are first created on the semiconductor NCH. The e^−^ in the CB of NCH reacts with H^+^ reducing it to H_2_. As also demonstrated by density functional theory (DFT) calculations in our previous publication,^14^ we confirmed that carbonates have a favorable electronic structure for the HER and strong adsorption for H atoms.

## Conclusions

In summary, a hierarchical mesoporous structure of nickel carbonate hydroxide hydrate (Ni_2_(CO_3_)(OH)_2_·4H_2_O) was successfully synthesized *via* the one-step hydrothermal route. The photophysical properties of NCH were thoroughly studied by experiments proving that NCH is suitable as a photocatalyst for hydrogen evolution. As a proof of concept, hierarchical NCH nanostructures were applied as a photocatalyst for photocatalytic hydrogen production for the first time and an H_2_ evolution rate of ∼10 μmol g^−1^ h^−1^ was obtained benefitting from the material structural features. Along with material innovation and in-depth study of the photocatalytic mechanism, this work is hoped to inspire a materials engineering route focused on metal carbonate hydroxide photocatalysts for sunlight hydrogen evolution.

## Author contributions

P. T.: conceptualization, investigation, methodology, writing – original draft. R. G.: supervision, writing – review & editing. T. Y.: investigation. M. Z.: investigation. S. A.: investigation. W. C.: writing – review & editing.

## Conflicts of interest

There are no conflicts to declare.

## Supplementary Material

MA-005-D3MA00977G-s001
