# Supplementary material for: Hierarchical nickel carbonate hydroxide nanostructures for photocatalytic hydrogen evolution from water splitting
Source: Mater Adv. 2024 Feb 27;5(7):2968–73. doi: 10.1039/d3ma00977g (PMC10986478; doi:10.1039/d3ma00977g)
Supplement: MA-005-D3MA00977G-s001 [file MA-005-D3MA00977G-s001.pdf]

Electronic Supplementary Material (ESI) for Materials Advances.
This journal is © The Royal Society of Chemistry 2024

Hierarchical Nickel Carbonate Hydroxide Nanostructures for Photocatalytic Hydrogen Evolution from Water Splitting

Parisa Talebi,[*a] Rossella Greco,[*a] Takashi Yamamoto,[b] Mahdiyeh Zeynali,[c] Saeid Asgharizadeh [c] and Wei Cao [a]

[a] Nano and Molecular Systems Research Unit, University of Oulu, FIN-90014, Finland

[b] Department of Science and Technology, Tokushima University, Tokushima 606-8506, Japan

[c] Faculty of Physics, University of Tabriz, Tabriz 5166616-471, Iran

Corresponding authors: rossella.greco@oulu.fi, parisa.talebi@oulu.fi

Experimental section

Synthesis of Nickel hydroxide

Nickel hydroxide was synthesized by hydrothermal Method. Typically, 2.5 mmol nickel nitrate hydrate ($Ni(NO_3)_2.6H_2O$) (99 %, Sigma-Aldrich) was solved in 200 mL deionized water. 40 mL ammonia 25% (99 %, Sigma-Aldrich) was added to it. 5 mmol of $NaHCO_3$ was solved in 50 mL DI water and immediately mixed with the previous solution. The solution was transferred to a Teflon-lined stainless-steel autoclave, which was hydrothermally treated at 120 °C overnight. After the autoclave was cooled down naturally to room temperature, the sample was taken out, washed with deionized water several times, and dried under vacuum overnight.

Characterization

XRD patterns were collected on a Rigaku Smart Lab system equipped with a five-axis θ-θ goniometer and 1D solid-state detector and scintillator using Co-Kα (λ=1.79 Å, 40 kV, 135 mA) radiation. SEM was carried out on Zeiss Sigma-field emission scanning electron microscopy (FESEM). Transmission electron microscopy (TEM) was performed on JEOL JEM-2200FS EFTEM/STEM equipped with an energy dispersive spectrometer (EDS). The X-ray absorption near edge structure (XANES) and extended X-ray absorption fine structure (EXAFS) spectra at the Ni K-edge were collected with an R-XAS Looper (Rigaku, Japan) equipped with a curved Si(400) monochromator crystal in transmission mode at room temperature. Data reduction was performed with the REX2000 program. The phase shift and background amplitudes were calculated using the FEFF8.4 program.[1] X-ray photoelectron spectroscopy (XPS) was carried out on a Thermo Fisher Scientific ESCALAB 250Xi XPS System with a monochromatic Al-Kα source. The binding energy was calibrated using the C 1s photoelectron peak at 284.8 eV as a reference and Au was used as a sample holder for the XPS measurements. N_2 adsorption-desorption isotherms were measured at −196 °C using a Micrometrics ASAP 2020 apparatus to determine specific surface areas, pore sizes,

and pore volumes of the materials. Before N_2 adsorption, the samples were degassed at 100 °C under vacuum for 12 h. The Brunauer-Emmett–Teller Barret-Joyner-Halenda (BET-BJH) methods were used for the calculations. DRIFT spectroscopy was conducted using a Bruker Vertex v80 device equipped with a Harrick Praying Mantis DRIFT cell with an MCT detector within an interval ranging from 4000 to 400 cm^{-1}. The spectrum was recorded with a resolution of 4 cm^{-1} using 100 signal-averaged scans. UV-visible (UV-vis) absorbance spectra were obtained using a Shimadzu UV-2600 spectrophotometer in the absorption range of 300 to 1000 nm. CS analysis was made using a Leco CS230 analyzer.

The photocatalytic hydrogen evolution activity of NCH was measured using a quartz bottle with dimensions 90 mm × 35 mm (height × diameter) and a total volume of ~ 68 mL. In a typical run, the catalyst (5 mg) was suspended in 25 mL of DI water followed by sonication. LED light source equipped with a magnetic stirrer in the Perfect Light PCX50B photo reactor was used as excitation. The solution was exposed to light for 2 h at room temperature. To measure the amount of the produced H_2, an Agilent 8860 gas chromatograph (GC) equipped with a split/splitless inlet, a TCD detector, and a capillary column CP-Molsieve 5 Å 25 m × 0.53 mm × 50 μm was used. All measurements were carried out without any cocatalyst and electron/hole sacrificial agent. To carry out the stability tests, the catalyst solution was illuminated for 3 cycles with each cycle of 2 h.

DFT details

The equilibrium geometries and energies of different possible phases of $Ni_2CO_3(OH)_2$ were calculated using the first-principles method based on DFT and implemented in the Quantum espresso Package.[2] We employed the generalized gradient approximation with the Perdue–Burke–Ernzerhof (PBE)[3] to describe the exchange-correlation functional. In order to improve the description of the highly localized Ni-d and O-p orbitals, we employed the so-called GGA+U correction scheme, where we used a Hubbard parameter U_d ranging from 4 to 11 eV for Ni-d orbitals and Up value ranges from 5 to 11 eV for O-p orbitals to reproduce the experimental band gap of Ni oxide (NiO)[4]. In our

calculations, the *k*-mesh based on the Monkhorst-Pack was used and cut-off energy was set to 24 × 9 × 6 and 475 eV for NiCH. Convergence criteria for the total energy and the ionic relaxation were 10^{-8} eV/atom and 10^{-4}eV/Å, respectively.

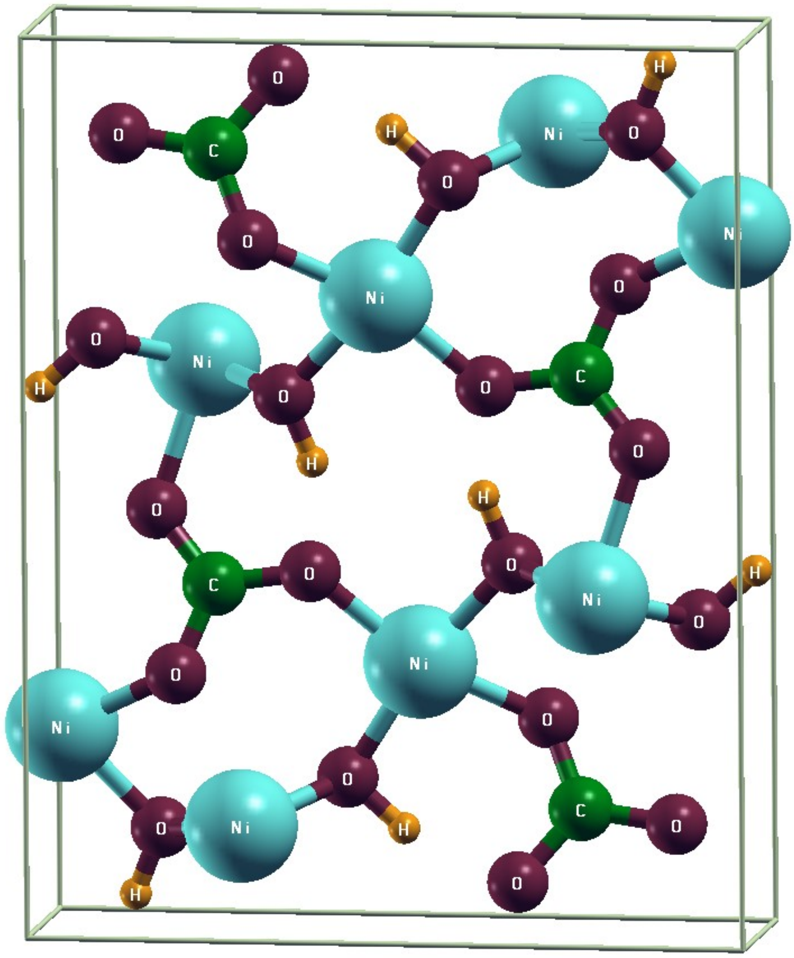

Figure S1. Side view of relaxed structure NCH by DFT.

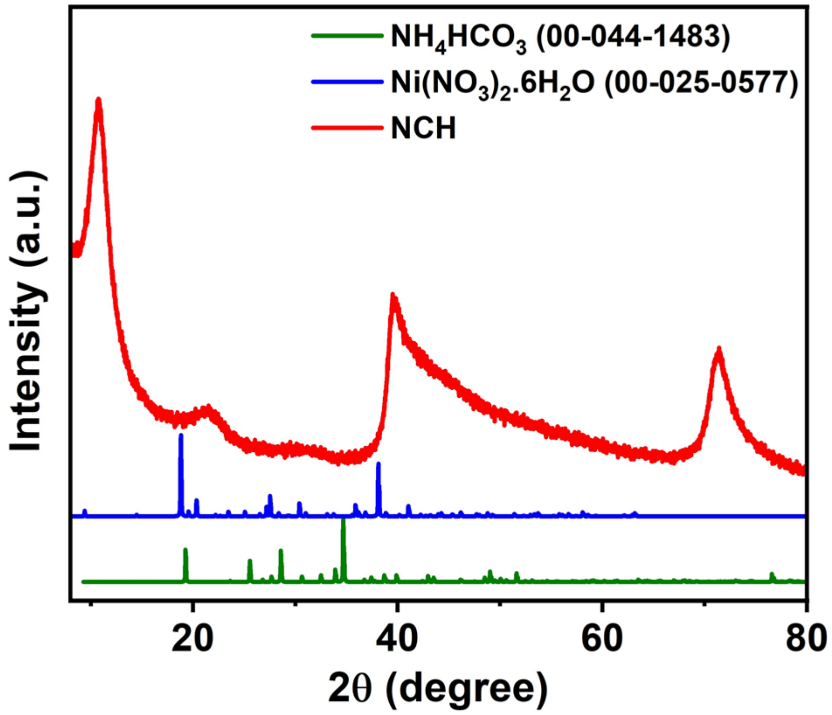

Figure S2. XRD pattern of NCH and precursors.

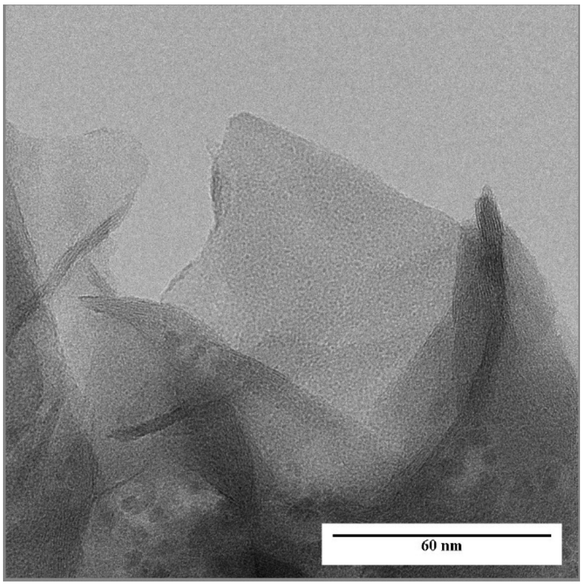

Figure S3. HRTEM image of NCH.

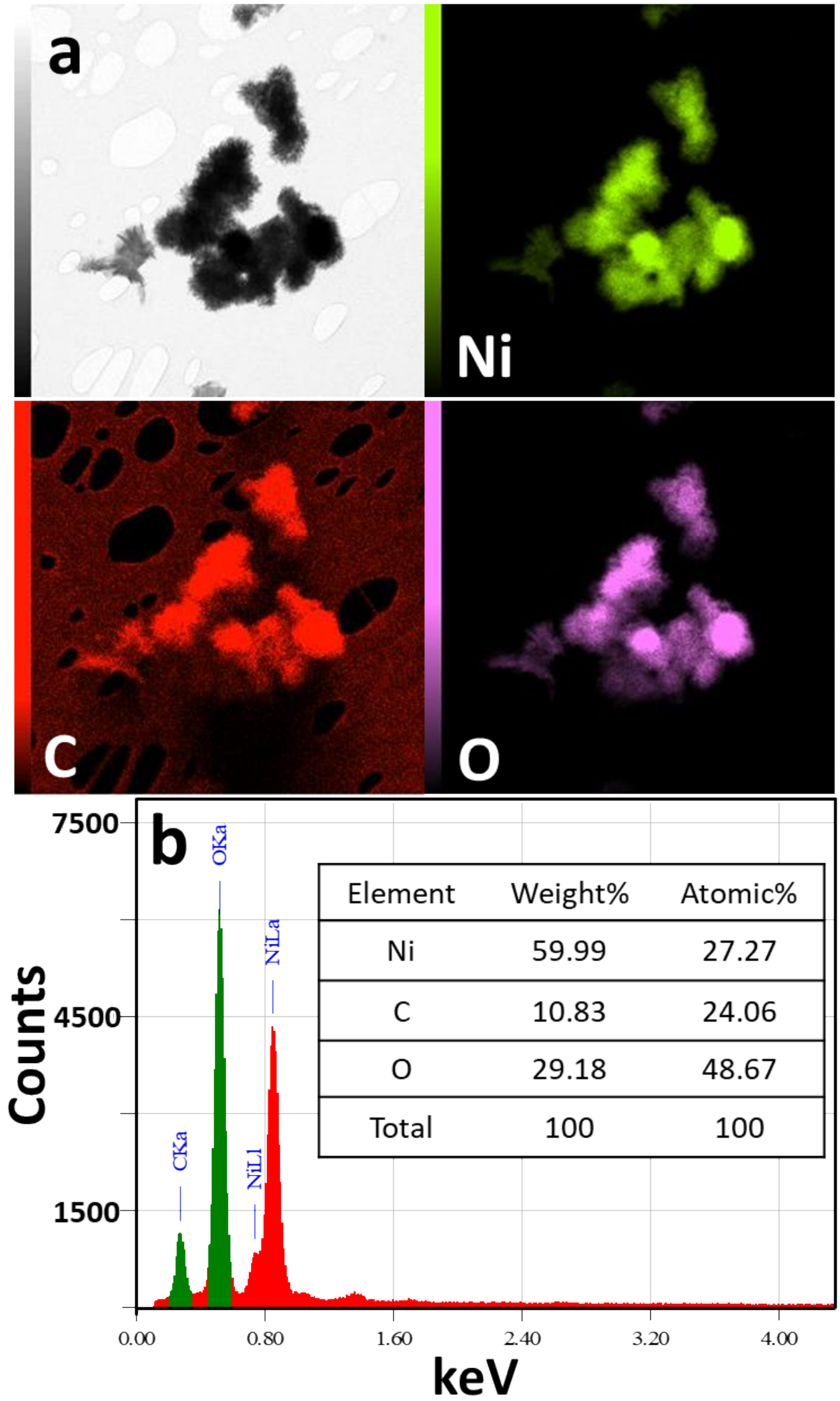

Element	Weight%	Atomic%
Ni	59.99	27.27
C	10.83	24.06
O	29.18	48.67
Total	100	100

Figure S4. (a) Elemental mapping images, and (b) EDS of NCH.

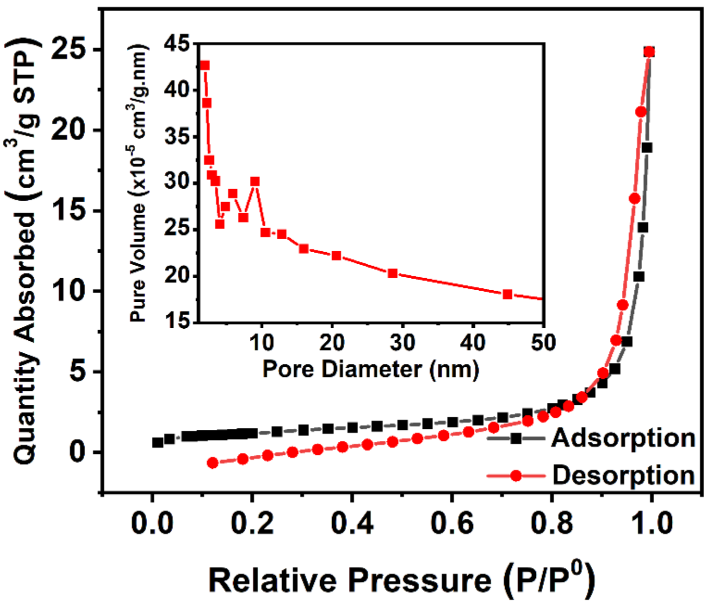

Figure S5. BET analysis of nitrogen adsorption-desorption isotherm and pore-size distribution curves (inserted image) of NCH.

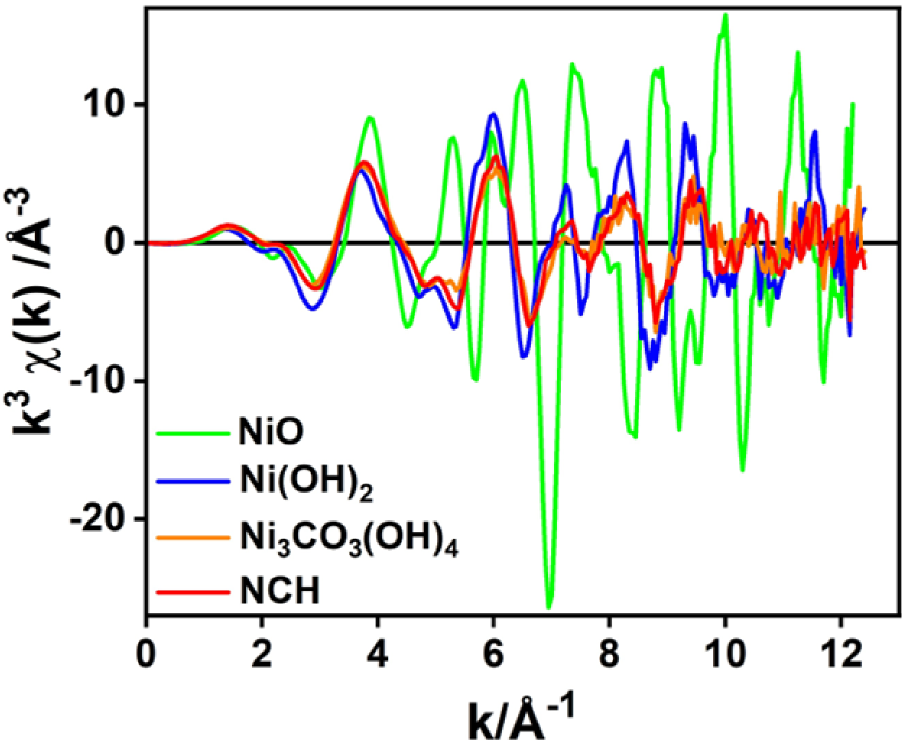

Figure S6. Ni K-edge EXAFS of NCH and commercial samples

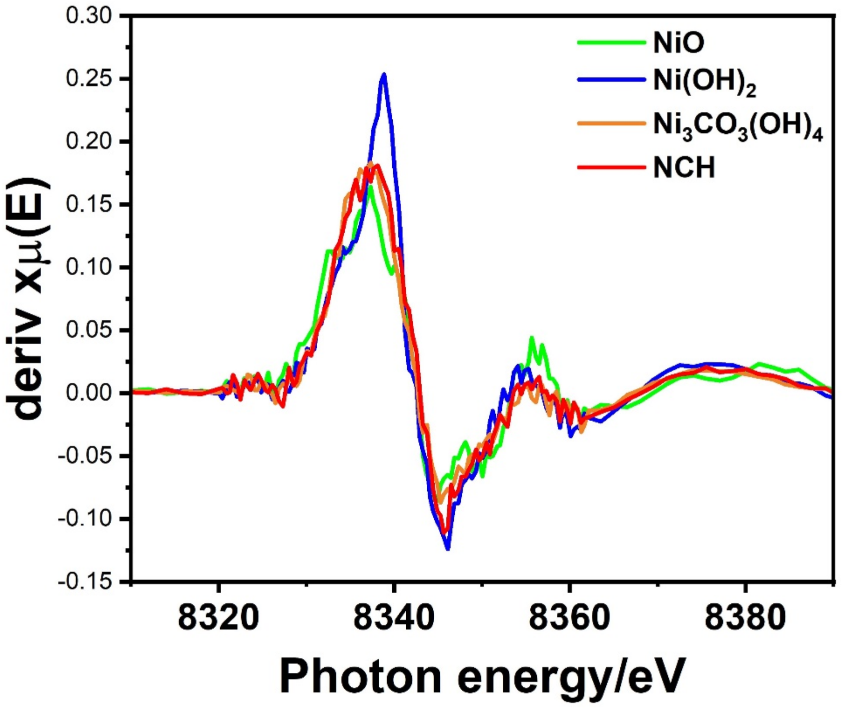

Figure S7. First derivate spectra of all samples

Table S1. EXAFS fitting parameters of NCH and commercial samples extracted from the Ni K-edge.

Sample	Shell	CN [a]			r [b] /Å			Ds [c] /Å		
NCH	Ni-O	5.5	±	*1.0*	2.04	±	*0.01*	0.098	±	*0.024*
	Ni-Ni	5.1	±	*1.5*	3.10	±	*0.01*	0.097	±	*0.025*
$Ni_3CO_3(OH)_4 \cdot 4H_2O$	Ni-O	4.8	±	*0.8*	2.04	±	*0.01*	0.088	±	*0.023*
	Ni-Ni	4.6	±	*1.6*	3.12	±	*0.01*	0.105	±	*0.029*
$Ni(OH)_2$	Ni-O	5.2	±	*1.0*	2.06	±	*0.01*	0.082	±	*0.024*
	Ni-Ni	6.1	±	*1.7*	3.13	±	*0.01*	0.082	±	*0.024*
NiO	Ni-O	4.3	±	*1.6*	2.09	±	*0.02*	0.081	±	*0.019*
	Ni-Ni	11.0	±	*2.4*	2.95	±	*0.01*	0.081	±	*0.019*

[a]Coordination number, [b]Interatomic distance, [c]Debye-Waller factor

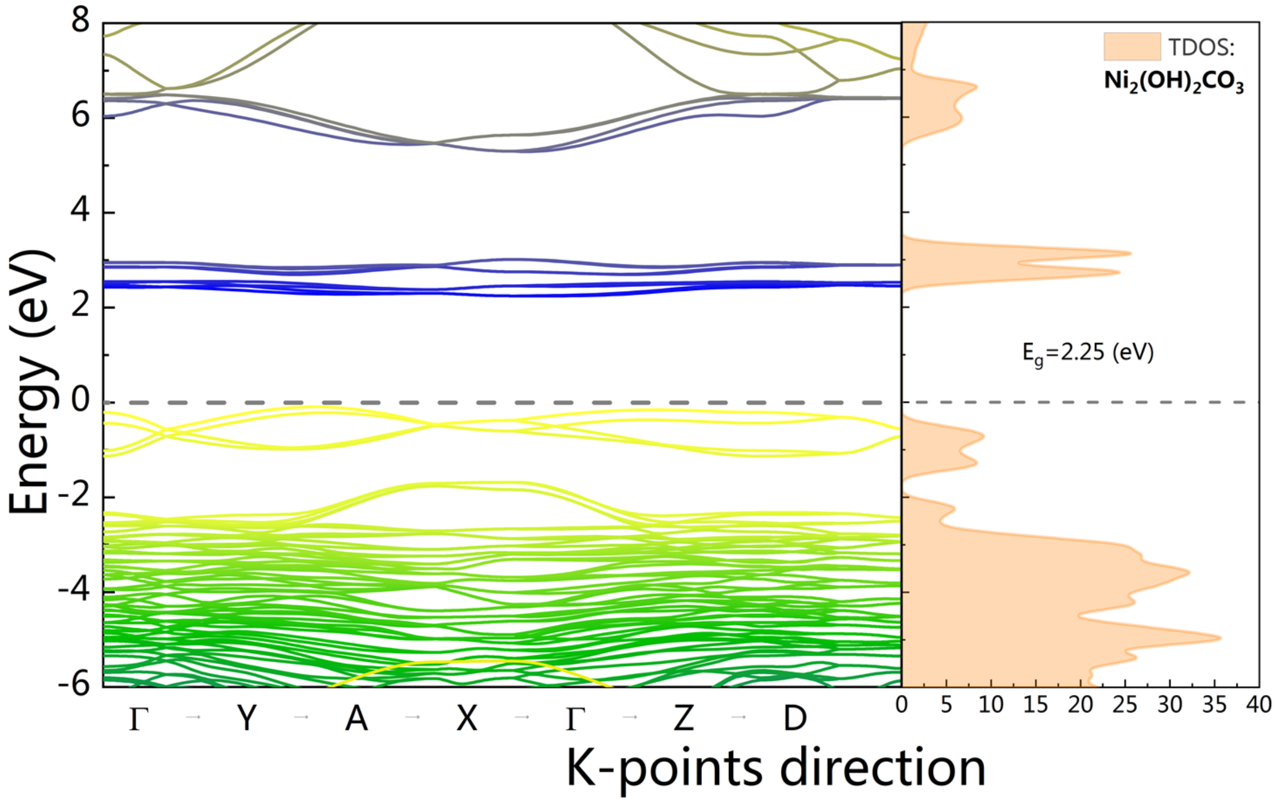

Figure S8. Electronic structure and DOS of NCH.

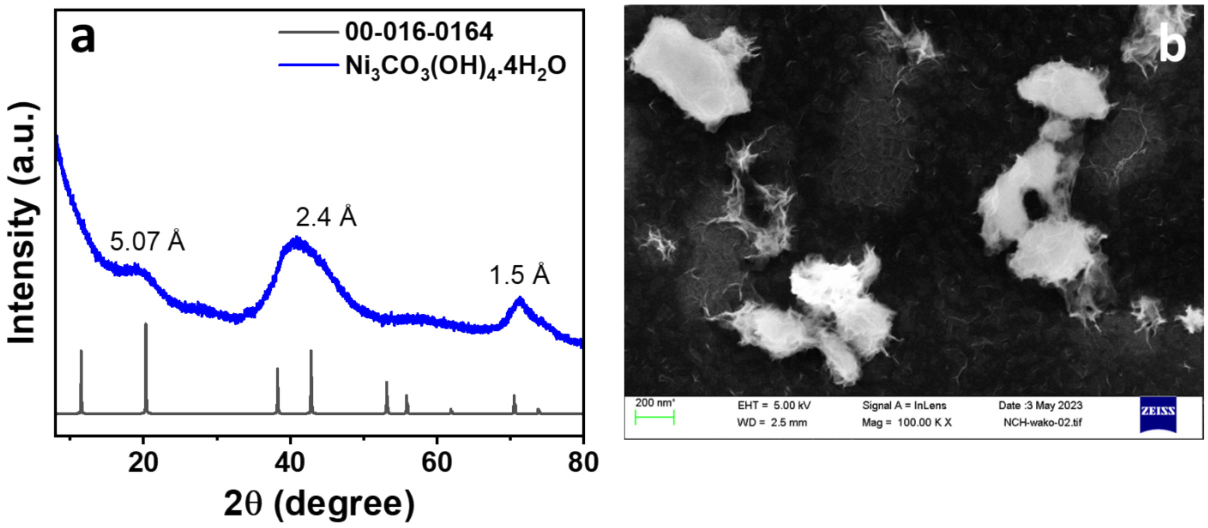

Figure S9. (a) XRD, and (b) SEM image of commercial $Ni_3CO_3(OH)_4.4H_2O$.

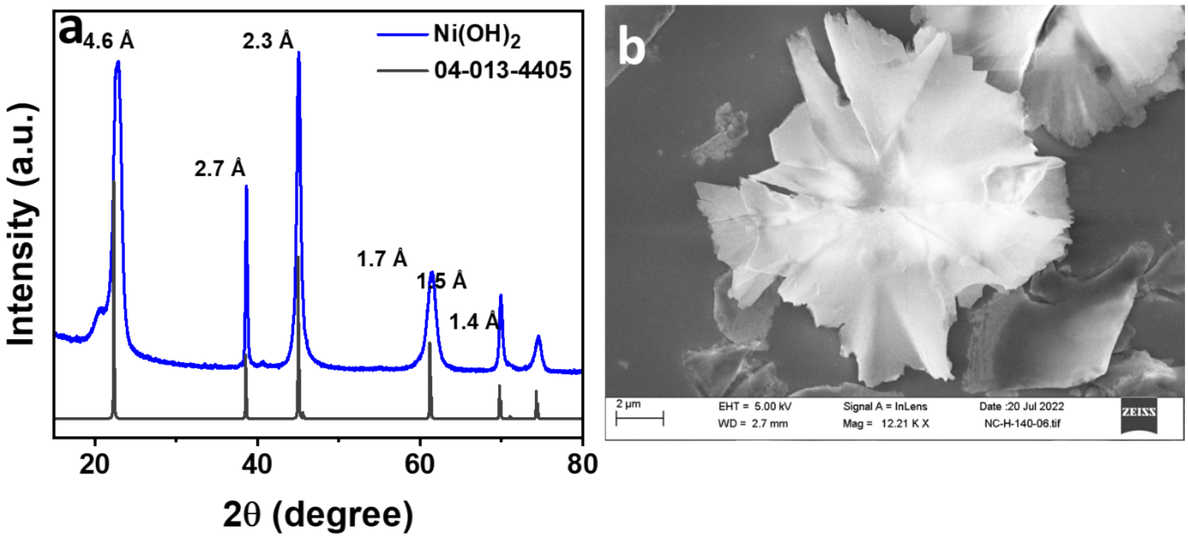

Figure S10. (a) XRD, and (b) SEM image of $Ni(OH)_2$.

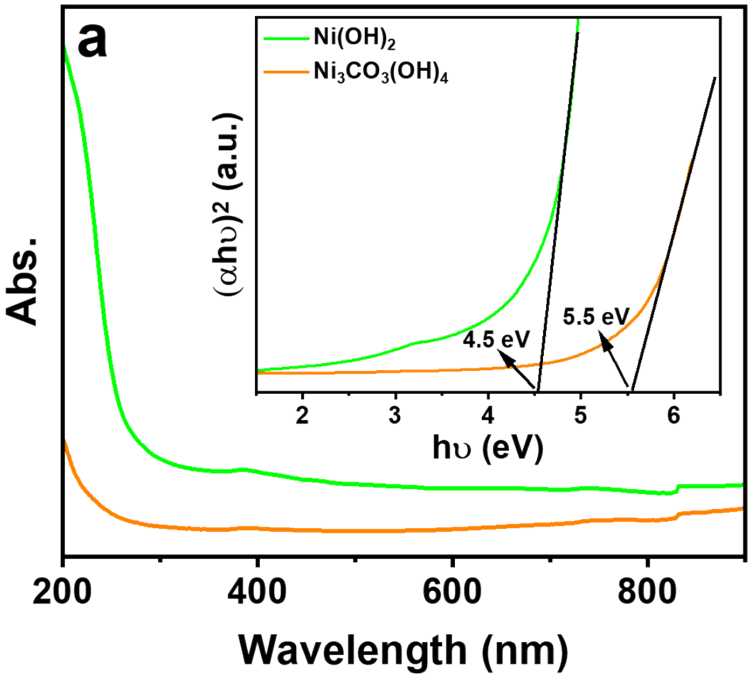

Figure S11. UV-vis absorbance spectra and Tauc plot (inserted image) of $Ni(OH)_2$ and $Ni_3CO_3(OH)_2$.

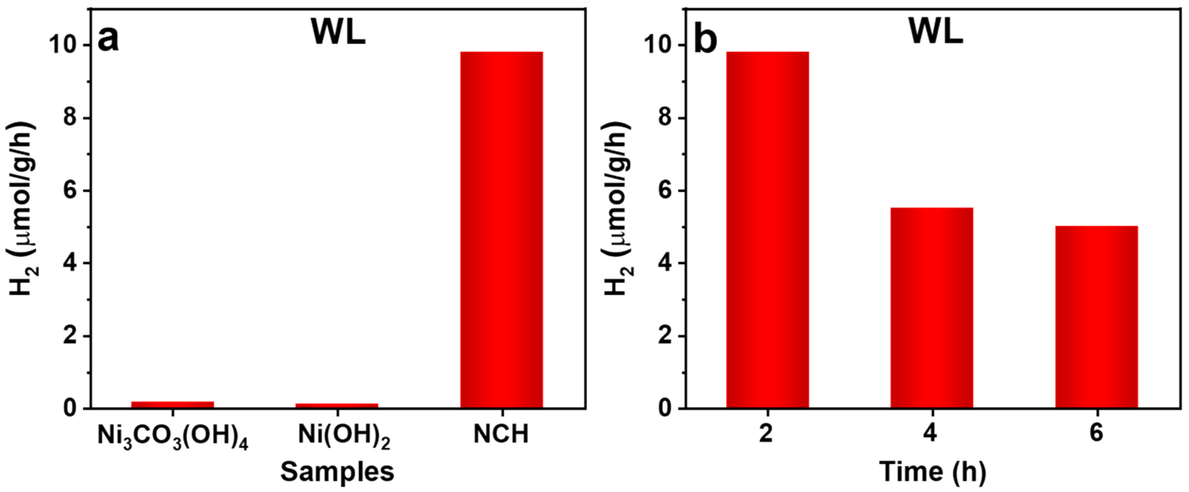

Figure S12. (a) HER of commercial $Ni_3CO_3(OH)_4.4H_2O$, $Ni(OH)_2$, and NCH and (b) stability tests of NCH under white light irradiation.

References

1. A. L. Ankudinov, B. Ravel, J. J. Rehr and S. D. Conradson, *Phys. Rev. B*, 1998, **58**, 7565.

2. G. Kresse and D. Joubert, *Phys. Rev. B*, 1999, **59**, 1758−1775.

3. J. P. Perdew, K. Burke and M. Ernzerhof, *Phys. Rev. Lett.*, 1996, **77**, 3865−3868.

4. E.S. Goh, J.W. Mah and T.L. Yoon, *Comput. Mater. Sci.*, 2017, **138**, 111–116.
